# PERSISTENT PLACOID MACULOPATHY: Long-term Outcomes and Prognostic Factors

**DOI:** 10.1097/IAE.0000000000004914

**Published:** 2026-06-26

**Authors:** Claire Seppey, Eirini Kaisari, Mickael Anjou, Chiara M. Eandi, Aude Ambresin, Antoine P. Brézin, Yan Guex-Crosier, Florence Hoogewoud

**Affiliations:** * Department of Ophthalmology, Fondation Asile des Aveugles, Hôpital Ophtalmique Jules-Gonin, Université de Lausanne, Lausanne, Switzerland;; † Ophtalmopôle de Paris, Hôpital Cochin, Department of Ophthalmology, Université Paris Descartes, Paris, France;; ‡ Department of Surgical Science, University of Torino, Torino, Italy; and; § Swiss Visio Retina Research Center, Swiss Visio Montchoisi, Lausanne, Switzerland.

**Keywords:** acute posterior multifocal placoid pigment epitheliopathy, chorioretinitis, choroidal neovascularization, macular atrophy, multimodal imaging, placoid lesions, persistent placoid maculopathy, treatment, uveitis, visual prognosis, white dots syndrome

## Abstract

**Purpose:**

To evaluate the long-term visual outcomes, recurrence, complication rate, and systemic associations of persistent placoid maculopathy. We also aimed to identify predictors of final visual acuity.

**Methods:**

This study combined a retrospective multicenter case series from three tertiary referral centers with a systematic review of the literature. Inclusion criteria were bilateral placoid macular lesions with characteristic multimodal imaging findings and a minimum follow-up of 23 months. Visual acuity, systemic associations, complications, recurrence, and treatments were assessed.

**Results:**

Twenty-three patients (46 eyes) were included, six patients from our centers and 17 patients from the literature. The median follow-up was 3.75 years. Severe visual loss (<20/100) at final follow-up was observed in one third of eyes. Choroidal neovascularization occurred in 65.2% of eyes and recurrence in 21.7%. Systemic cardiovascular comorbidities were common (87.5%), and two patients presented cerebral vasculitis. Systemic treatment was significantly associated with better final visual acuity (*P* = 0.014).

**Conclusion:**

Persistent placoid maculopathy presents a high complication rate and poor visual prognosis. Systemic treatment was significantly associated with better final visual acuity in our study.

## Abstract 2

Supplemental Digital Content is Available in the Text.

Persistent placoid maculopathy is a rare condition with high rates of complications and poor visual outcomes. In this multicenter retrospective study, systemic treatment was significantly associated with better final visual acuity.

Persistent placoid maculopathy (PPM) is a rare bilateral maculopathy first described in 2006 in a series of six cases.^1^ Since then, approximately 60 cases have been reported in the literature. Initially thought to primarily affect Whites, a recent study identified the disease in an Asian cohort.^2^

Despite its recent identification, PPM has well-defined imaging characteristics. In the early stages, macular whitish plaque-like lesions appear, distinct from the optic disc. Fluorescein angiography reveals a hypofluorescent lesion in the early phase with late staining. Indocyanine green angiography (ICGA) consistently shows hypofluorescence throughout all phases. Optical coherence tomography (OCT) highlights outer retinal and retinal pigment epithelium changes, including outer nuclear layer hyperreflectivity and loss of the external limiting membrane and ellipsoid zone. The choriocapillaris appear hyporeflective and thickened, corresponding to a flow signal void on OCT-angiography (OCTA) and hypofluorescent lesions on ICGA (Figure 1).^3–8^

**Fig. 1.**
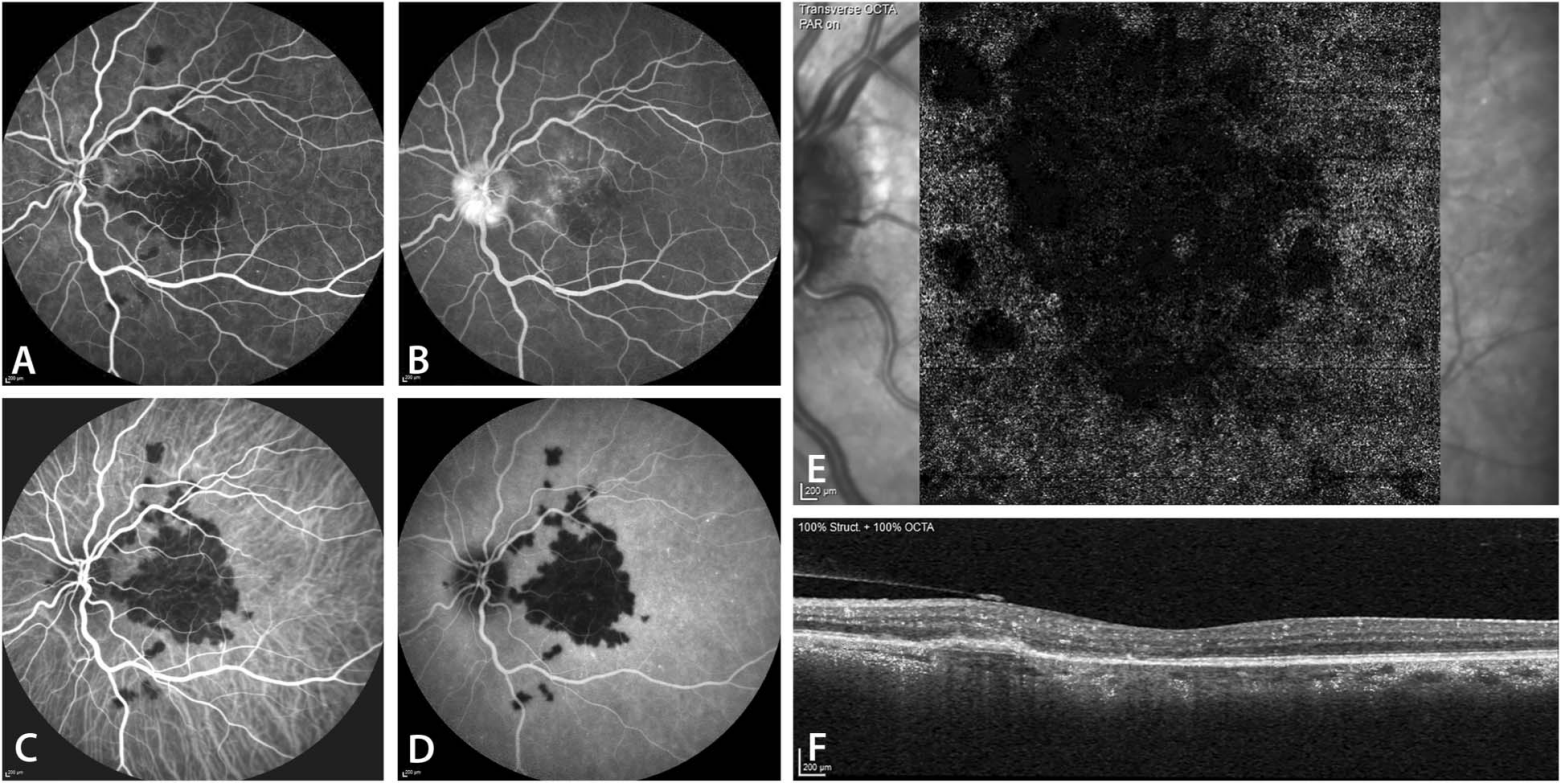
Multimodal imaging in persistent placoid maculopathy: **A.** early-phase fluorescein angiography (FA) (38 seconds) showing hypofluorescent lesions. **B.** Late-phase FA (10:55 minutes) showing resolution of the hypofluorescence and late staining of the lesions. **C** and **D.** Hypofluorescence at all time on the ICG angiography (C. 2:20 minutes, D. 11:37 minutes). **E** and **F.** Hyporeflectivity of the choriocapillaris corresponding to a void of flow signal within the choriocapillaris on OCTA. Because of space limitations, only the left eye is shown, the condition is symmetric in the right eye.

PPM shares certain characteristics with acute posterior multifocal placoid pigment epitheliopathy (APMPPE) and serpiginous choroiditis (SC), yet it has distinct morphologic and demographic features that help in its differentiation: although placoid lesions in APMPPE are more scattered throughout the retina and typically affect younger patients, PPM lesions are confluent, located in the posterior pole, and tend to occur in older individuals. In addition, the distinctive hyperfluorescent border commonly seen in SC is absent in PPM.^9^ SC lesions are classically juxtapapillary and serpiginous, whereas PPM lesions are macular and plaque-like.

The exact cause of PPM has not been elucidated, but inflammation is believed to take an important part in the etiology.^6,10^ Because of the lack of data on the long-term prognosis of PPM, the absence of randomized studies, and the rarity of PPM, there is no consensus on the treatment.

The aim of this study is to define the long-term outcomes of PPM by determining the recurrence rate, complication rate, and visual outcome in patients reported in the literature combined to our own cases. For the purpose of this study, long-term follow-up refers to a minimum of 23 months, with an observed median follow-up of more than 3 years. In addition, we sought to identify associated systemic factors that may help explain the disease pathophysiology.

## Material and Methods

This study was designed as a retrospective observational analysis combining a multicenter case series with a systematic review of the literature. Patients from three tertiary referral centers (Jules-Gonin Eye Hospital, Lausanne; Cochin Hospital, Paris; and Swiss Visio Montchoisi, Lausanne) were included, together with previously published cases, meeting the same inclusion criteria.

Patients presenting bilateral placoid macular lesions with characteristic imaging properties (hypofluorescent macular lesion at all time on ICGA, hypofluorescent lesion with late staining on fluorescein angiography, and outer retinal changes on OCT) (Figure 1) were included. A minimum follow-up of 23 months was required as an inclusion criterion, whereas the actual follow-up duration was substantially longer and is reported in the results. Previous ancillary testing to exclude alternative diagnosis including syphilis (*Treponema pallidum* enzyme-linked immunosorbent assay) and tuberculosis (Tspot or QuantiFERON test) was required.

The study was conducted in compliance with the principles of the Declaration of Helsinki and was approved by the Institutional Review Board (CER-VD no. 2018-02161 for the Swiss patients and IRB00008855, Société française d'ophtalmologie, IRB#1 for the French patients).

In addition, a comprehensive review of the literature was performed on PubMed, Cochrane Library, GoogleScholar, Web of Science, up to March 2025. The search terms included “persistent placoid maculopathy,” “placoid maculopathy,” and “persistent placoid chorioretinitis.” References from relevant articles were evaluated for additional papers. Published cases were included if sufficient clinical, imaging, and follow-up data were available and if the diagnostic criteria matched those applied to patients from our centers. To minimize duplicate reporting, cases were cross-checked based on institution, authorship, patient demographics, clinical presentation, and imaging characteristics. When overlap was suspected, only the most complete report was retained. A total of 112 papers were screened, of which 33 were considered relevant to our study, and 10 reported cases eligible for inclusion.

Data collection included demographic data, best-corrected visual acuity (BCVA) at baseline and during follow-up, systemic comorbidities, and treatment regimens. BCVA scores were reported in Snellen scale and were converted to LogMAR for statistical analysis. Complications such as disciform macular scar (or macular atrophy) and choroidal neovascularization (CNV) as well as recurrences were also recorded.

Published data along with our own data were collected and statistics were applied (Excel, SAS). Because of the scarcity of PPM, there are no randomized clinical trials available and the results found in the literature are not standardized, so that meta-analysis was not possible. For descriptive statistics, we used median values with interquartile range (IQR) for the description of patient characteristics and outcomes.

We performed normality tests (Shapiro–Wilk, Kolmogorov–Smirnov) on both the residuals from the linear regression model and the final visual acuity (LogMar) variable. The results show that the final visual acuity does not follow a normal distribution. Conversely, the residuals approximatively satisfy the normality assumption, allowing us to use a linear regression model for statistical analysis. We used quantile regression model to confirm statistical significance.

## Results

Table 1 summarizes separately the visual outcomes, complication rates, and treatments of patients from our local cohort as well as those reported in the literature.

**Table 1.** Clinical characteristics, treatment, and outcomes

Patient Number	Patient Source	Age (years)	Sex	Cardiovascular Risk Factor	Eye	Initial VA (Snellen)	Final VA (Snellen)	Follow-up (months)	Choroidal Neovascularization	Disciform Scar	Anti-inflammatory Treatment	Recurrence
1		72	M	Yes	OD	20/20	20/20	87	No	No	sCS + IMT	Yes
					OS	20/20	20/32		Yes	No	sCS + IMT	Yes
2		59	M	Yes	OD	20/20	20/20	122	Yes	No	sCS + IMT	Yes
					OS	20/1,250	20/25		Yes	No	sCS + IMT	Yes
3		69	M	Yes	OD	20/100	20/200	24	No	No	sCS	No
					OS	20/400	20/100		No	No	sCS	No
4		49	M	Yes	OD	20/63	20/25	24	No	Yes	sCS	No
					OS	20/25	20/25		No	Yes	sCS	No
5		65	F	Yes	OD	20/20	20/20	45	No	Yes	None	No
					OS	20/20	20/32		Yes	Yes	Local CS	No
6		58	M	Yes	OD	20/20	20/16	46	No	No	sCS + IMT	Yes
					OS	20/20	20/16		No	No	sCS + IMT	Yes
Total local cohort		*63.4 (9.4)	M 5/6 (83.3%)	6/6 (100%)		†20/20 (20/1,250–20/20)	†20/25 (20/200–20/16)	†47 (24–122)	4/12 (33.3%)	4/12 (33.3%)		6/12 (50%)
7	Golchet et al	68	M	Yes	OD	20/20	20/200	76	Yes	Yes	sCS	No
					OS	20/1,250	20/400		Yes	No	sCS	No
8	Golchet et al	50	F	Yes	OD	20/25	20/32	26	Yes	No	Local + sCS	No
					OS	20/25	20/25		Yes	No	Local + sCS	No
9	Golchet et al	53	M	Yes	OD	20/25	20/80	34	Yes	Yes	Local CS	No
					OS	20/20	20/400		Yes	Yes	None	No
10	Golchet et al	59	M	Yes	OD	20/25	20/200	245	Yes	Yes	None	No
					OS	20/50	20/320		Yes	Yes	None	No
11	Waisbren et al	60	M	NA	OD	20/20	20/32	48	Yes	No	None	Yes
					OS	20/32	20/200		Yes	No	None	No
12	Parodi et al	60	M	No	OD	20/800	20/800	24	Yes	Yes	None	No
					OS	20/32	20/20		Yes	No	None	No
13	Nika et al	55	M	Yes	OD	20/125	20/250	24	Yes	No	sCS + IMT	Yes
					OS	20/320	20/800		Yes	No	sCS + IMT	No
14	Gascon et al	61	M	No	OD	20/25	20/25	36	No	Yes	None	No
					OS	20/50	20/40		Yes	No	None	No
15	Klufas et al	42	F	NA	OD	20/50	20/63	24	Yes	No	sCS	No
					OS	20/40	20/32		Yes	No	sCS	No
16	Li et al	62	NA	NA	OD	NA	NA	26	No	No	NA	No
					OS	NA	NA		No	No	NA	No
17	Li et al	51	NA	NA	OD	NA	NA	58	No	No	NA	No
					OS	NA	NA		No	No	NA	No
18	Li et al	49	NA	NA	OD	NA	NA	121	Yes	No	NA	No
					OS	NA	NA		Yes	No	NA	No
19	Li et al	66	NA	NA	OD	NA	NA	50	No	No	NA	No
					OS	NA	NA		No	No	NA	No
20	Kolmeyer et al	59	M	Yes	OD	20/20	20/20	23	No	No	sCS + IMT	Yes
					OS	20/20	20/25		Yes	No	sCS + IMT	Yes
21	Levin et al	65	M	Yes	OD	20/40	20/32	26	Yes	Yes	sCS + IMT	No
					OS	20/20	20/20		Yes	No	sCS + IMT	No
22	Yu Cheng et al	78	M	Yes	OD	20/63	20/200	48	Yes	Yes	None	No
					OS	20/50	20/50		Yes	No	None	No
23	Yu Cheng et al	57	M	NA	OD	20/63	20/1,250	87	Yes	No	None	No
					OS	20/50	20/50		Yes	No	None	No
Total literature		*57.3 (7.2)	11/13 (84.6%)	8/11 (72.7%)		†20/32 (20/1,250–20/20)	†20/50 (20/1,250–20/20)	†34 (23–245)	26/34 (76.5%)	9/34 (26.5%)		4/34 (11.8%)
Total cohort		*59.4 (8.3)	16/19 (84.2%)	14/17 (82.4%)		†20/25 (20/1,250–20/20)	†20/32 (20/1,250–20/16)	†45 (23–245)	30/46 (65.2%)	13/46 (28.2%)		10/46 (21.8%)

* Mean (SD).

† median (range).

M, male; F, female; NA, not available; OD, right eye; OS, left eye; sCS, systemic corticosteroids; IMT, immunosuppressive therapy (methotrexate, mycophenolate mofetil, azathioprine, cyclophosphamide).

### Demographics

We included a total of 23 patients in our analysis: three patients from Jules-Gonin Eye Hospital, two patients from Cochin Hospital, one patient from Swiss Visio Montchoisi, and 17 patients from the literature review.^1–4,6,8,11–15^ The median age at diagnosis was 59 years (range 42–78, IQR 11). Male gender was reported in 16/19 patients (84.2%, four patients missing).

### Follow-up

The median follow-up time of the whole cohort was 3.75 years (range 23–245 months, IQR 42), 3.91 years (range 24–122 months) for the local cohort and 2.83 years (range 23–245 months) for the literature cohort.

### Systemic Associations

Medical history and systemic conditions were available in 16 patients. Diabetes/prediabetes was present in five patients (31.3%), hypertension in eight patients (50%), stroke or coronary artery disease in three patients (18.8%), and smoking in four (25%). They were two patients without cardiovascular risk factors (12.5%).

In addition, two patients (both from our local cohort) had symptomatic cerebral vasculitis.

### Visual Symptoms and Baseline Visual Acuity

Visual symptoms at presentation were reported in 22 (73.3%) of the 30 eyes with known data. The most common symptom was decreased visual acuity, observed in 20 eyes (90.9%), followed by photopsias in two eyes (9%). Eight eyes (26.6%) were asymptomatic at presentation.

### Visual Acuity at Baseline and During the Follow-up

Visual acuity data were available for 38 eyes. The median initial visual acuity was 20/25 (range 20/20–20/1,250) and decreased to 20/32 at the final follow-up (range 20/16–20/1,250).

Severe visual loss (≤20/100) in 29.0% of patients, whereas 31.6% retained a final BCVA greater than 20/40 Snellen.

### Complications and Recurrences

Choroidal neovascularization was detected in 30 eyes (65.2%), and disciform scars were present in 13 eyes (28.2%). Both CNV and disciform scars developed in nine eyes (19.6%), whereas 12 eyes (26.1%) showed neither.

**Fig. 2.**
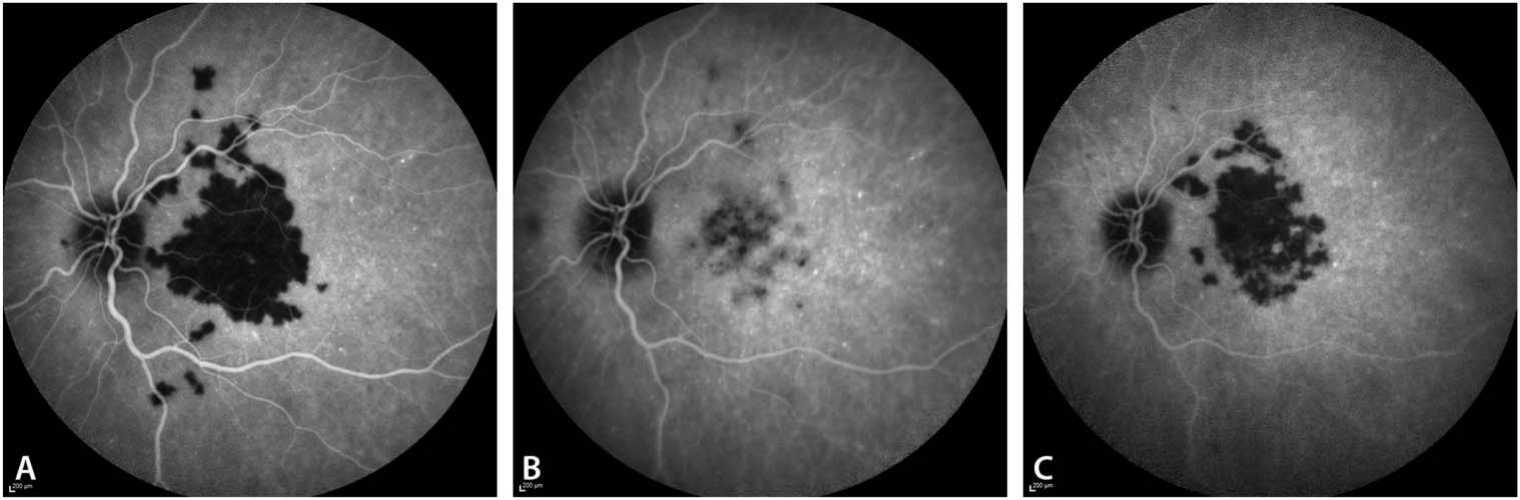
Recurrence in a case of persistent placoid maculopathy: **A.** initial presentation: hypofluorescent lesion on ICG angiography (11:37 minutes). **B.** Six-month follow-up: reduction of the hypofluorescent lesion under systemic azathioprine (14:31 minutes). **C.** Six years later: recurrence of the hypofluorescent lesion (11:26 minutes).

Recurrence was observed in 10 eyes (21.73%) (Figure 2). Out of those, seven eyes developed the recurrence while being systemically treated: either with systemic corticosteroids, immunosuppressive therapy, or both.

### Medical Therapy

Among the 38 eyes with known treatment regimens, 11 eyes (28.9%) received steroids alone (10 eyes with systemic prednisone and one eye with subtenon triamcinolone) and 12 eyes (31.57%) received a combination of steroids with immunomodulatory treatments (methotrexate, azathioprine, mycophenolate mofetil, or cyclosporine).

For the treatment of CNV, intravitreal anti-VEGF injections were given in 16 eyes (42.1%), and laser photocoagulation was performed in six eyes (15.8%). Three eyes (7.9%) received no treatment.

### Associations With Visual Outcomes

**Fig. 3.**
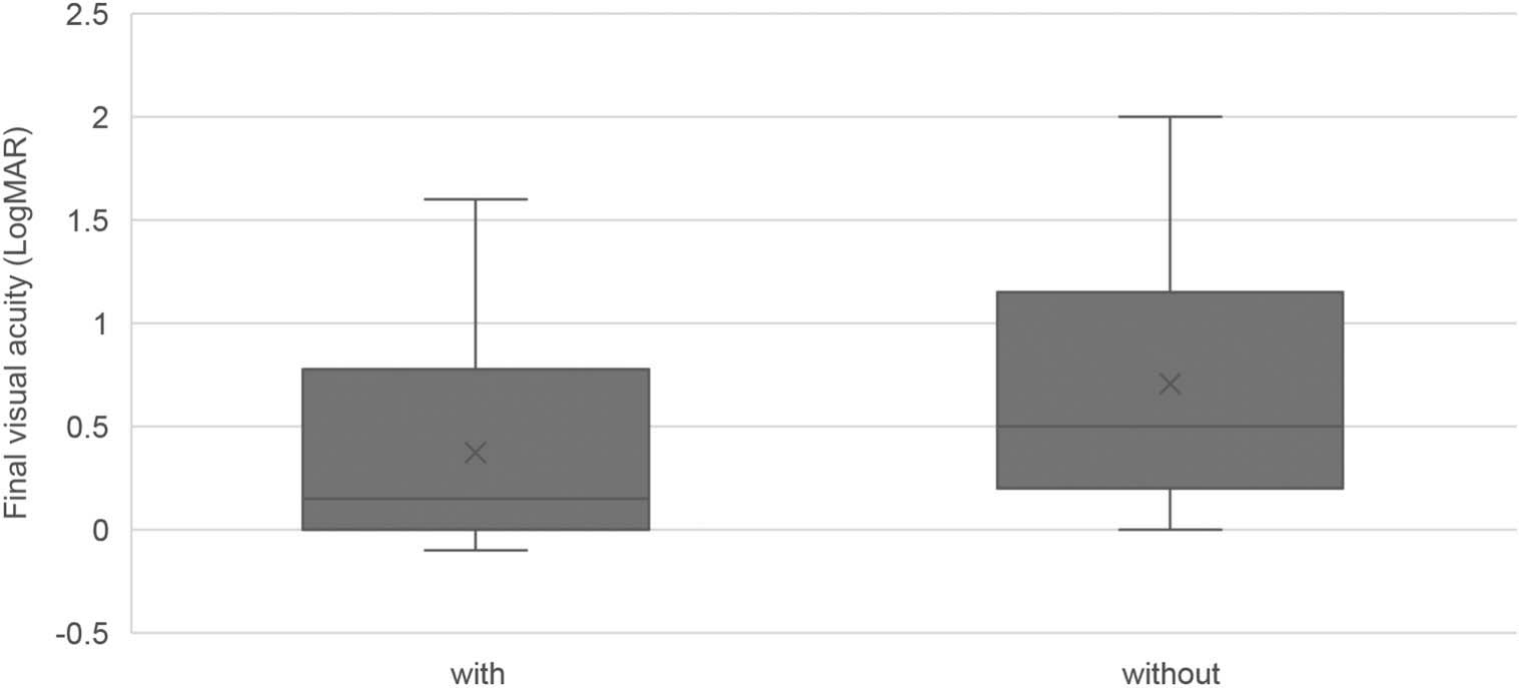
Final visual acuity (LogMAR) in patients with and without systemic treatment.

Final visual acuity was improved in patients treated systemically (Figure 3).

Classical linear regression model identified initial visual acuity (*P* < 0.01) and systemic treatment (*P* = 0.014) as significant predictive factor of the final visual outcome (Table 2).

**Table 2.** Classical Linear Regression of the Final Visual Acuity in LogMAR

Parameter	Estimate	*P*	95% Confidence Limits
Initial vision logMAR	0.584	0.001	0.271 to 0.897
Systemic treatment	−0.409	0.014	−0.728 to −0.090
Age	−0.007	0.475	−0.028 to 0.013
Sex	−0.240	0.303	−0.707 to 0.226

This result was confirmed with quantile regression model for initial visual acuity in all quantiles. Systemic treatment was significant at 5% confidence interval when looking at the quantile 0.5 (median), associated with better final vision. Similarly, the effect was also significant at the quantile 0.75, conversely to the lower quantile (0.25) (see **Supplementary Table 1**, **Supplemental Digital Content 1**, http://links.lww.com/IAE/C962). Age and gender were not significant.

## Discussion

This study is the first to evaluate the long-term clinical outcomes of PPM. The demographic characteristics of our study align with those reported in the literature, featuring a majority of male patients and a median age of 59 years. These demographics are similar to those observed in SC, with a mean age at time of diagnosis in Whites reported from 43.4 to 59.1 years.^16^

With a median follow-up of 3.5 years, approximately one third of affected eyes experienced severe visual loss. Complications were common, occurring in 73.9% of cases, highlighting the severe prognosis of PPM.

In our study, the most common complication was CNV. It affected 65.2% of eyes, a rate higher than typically reported in SC.^17^ The CNV rate was higher in the cases from the literature compared with our local cohort (76.5% vs. 33.3%). This discrepancy may be because of selection bias arising from differences in referral and recruitment patterns (i.e., enrollment through a uveitis clinic vs. a medical retina clinic), which could influence the observed stage and phenotype of the disease.

A similar selection bias might explain the observed difference in recurrence rates. Our local cohort showed a 50% recurrence rate, in contrast to the 11.8% recurrence rate found in the cases from the literature. We suspect that this variation could also be attributed to an underestimation of recurrences in previous reports. Detecting subclinical recurrences can be difficult with structural OCT, as it may lack the sensitivity to identify flow abnormalities in the choriocapillaris. OCTA or ICGA, being more sensitive imaging techniques, is better suited for detecting such abnormalities. We, therefore, recommend integrating OCTA into routine follow-up for these patients. Another factor influencing recurrence rates is the variation in follow-up duration. Notably, the latest recurrence in our series occurred after 6 years of quiescence, underscoring the importance of prolonged monitoring (Figure 2). Overall, the recurrence rate in our study appears lower than what is typically reported in SC.^17^ It is also worth noting that none of the patients with disciform scars experienced a recurrence of inflammation.

Our statistical analyses showed that systemic treatment was associated with better final BCVA, consistent with observations previously reported in the literature.^1,10^ This is significant at the quantile 0.5 and 0.75 but is not at the lower quantile 0.25. These results indicate that the effect of systemic treatment seems to be positive and more pronounced in patients with intermediate to good recovery and is unclear in patients affected more seriously, possibly because of limited room for improvement.

However, IMT did not prevent recurrences in some patients. Outcomes across studies are poorly defined, with variability in treatment choices, timing, dosage, duration, and therapy combinations, making meta-analysis difficult and the number needed to treat uncertain. Some studies suggest that high doses of corticosteroids may improve visual prognosis, and others recommend adding immunosuppressive therapy when hypoperfusion persists. However, in our study, none of the patients with disciform scars experienced recurrences. Therefore, the potential benefits of long-term steroid-sparing treatments in these patients remain uncertain.

Cardiovascular risk factors were identified in most patients with PPM (87.5%) suggesting a potential role in the disease's pathophysiology. It is hypothesized that PPM results from immune-triggered ischemia of the choriocapillaris.^5,6,10,18^ The condition shares many similarities with APMPPE; however, differences in outcomes may be linked to the systemic cardiovascular risk profile. The presence of diseased vessels in PPM could explain why APMPPE often progresses to recovery with capillary reperfusion, whereas PPM exhibits a higher incidence of macular scarring.

A shared feature between PPM and APMPPE is their association with cerebral vasculitis, which was observed in two of our patients. This condition can lead to severe life-threatening complications. As with APMPPE, it is recommended that any neurologic symptoms in patients with PPM should trigger prompt neuroimaging evaluation.^19,20^

There are limitations to our study and statistical analyses, which include the sample size, the variable follow-up duration, the limited control variables, and the retrospective nature of the study. Most data used for our statistical analyses were derived from the literature rather than from our local cohort, with an inherent risk of selection and publication bias. Although these initial statistical results are promising, the heterogeneity resulting from the combination of local and literature-derived cases may affect the reliability of the analyses and underscores the need for further research with larger sample sizes.

In conclusion, this study offers important insights into the long-term clinical outcomes of PPM, a rare condition with a reserved prognosis. Our findings emphasize that complications, particularly CNV, are common in PPM. The recurrence rate observed in our study highlights the need for long-term monitoring, as the condition can reactivate even after years of quiescence. Our statistical analyses support that final BCVA is better in patients treated systemically. However, the lack of consensus on the most effective treatment regimen calls for further research. Given the rarity of PPM and the current data limitations, future studies are essential to better understand the disease's pathophysiology and establish evidence-based treatment guidelines.

## Supplementary Material
