# Supplementary material for: PERSISTENT PLACOID MACULOPATHY: Long-term Outcomes and Prognostic Factors
Source: Retina. 2026 Jun 26;46(10):1740–7. doi: 10.1097/IAE.0000000000004914 (PMC13596190; doi:10.1097/IAE.0000000000004914)
Supplement: Supplementary file 1 [file retina-46-1740-s001.pdf]

Supplementary Table 1. Quantile regression of the final visual acuity in LogMAR

**Quantile 0.25**			
**Parameter**	**Estimate**	**95% confidence limits**	
Initial vision acuity (logMAR)	0.5449	0.0616	0.789
Systemic treatment	-0.1127	-0.2957	0.0623
Age	0.0028	-0.0253	0.0124
Sex	0.1068	-Infty	0.2395
**Quantile 0.5**			
Initial vision acuity (logMAR)	0.7240	0.4096	1.3431
Systemic treatment	-0.3697	-0.767	-0.0883
Age	-0.0132	-0.0188	0.0109
Sex	-0.1697	-0.3637	0.2172
**Quantile 0.75**			
Initial vision acuity (logMAR)	0.8107	0.3486	2.1115
Systemic treatment	-0.4834	-1.1501	-0.1419
Age	-0.0148	-0.0342	0.0112
Sex	-0.468	-0.8142	Infty
